# Inefficiencies identified in healthcare professional-to-patient handover practices for atrial fibrillation: a mixed-methods study in Brazil, China and Sri Lanka

**DOI:** 10.1136/bmjgh-2024-017517

**Published:** 2025-11-23

**Authors:** Tiffany E Gooden, Jingya Wang, Alessandra C Goulart, Sheron A Vethanayagam, Hao Wang, Ana C Varella, Elisabete Paschoal, Powsiga Uruthirakumar, Shribavan Kanesamoorthy, Shivany Shanmugathas, Hui Zhang, Jiaoyue Zhong, Mei Feng, Xiaojing Li, Mahesan Guruparan, Balachandran Kumarendran, Krishnarajah Nirantharakumar, Gregory Y H Lip, G Neil Thomas, Isabela M Bensenor, Yutao Guo, Surenthirakumaran Rajendra, Sheila Greenfield, Semira Manaseki- Holland, NIHR Global Health Group on Atrial Fibrillation Management members, Ajini Arasalingam

**Affiliations:** 1 Department of Applied Health Sciences, University of Birmingham, Birmingham, Birmingham, UK; 2 University of Sao Paulo, Sao Paulo, Brazil; 3 University of Sao Paulo University Hospital of Sao Paulo, Sao Paulo, São Paulo, Brazil; 4 University of Jaffna, Jaffna, Northern, Sri Lanka; 5 Chinese PLA General Hospital, Beijing, China; 6 Center for Clinical and Epidemiological Research, Universidade de Sao Paulo, Sao Paulo, Brazil; 7 Department of Marketing, University of Jaffna Faculty of Management Studies and Commerce, Jaffna, Northern, Sri Lanka; 8 Department of Cradiology, Jaffna Teaching Hospital, Jaffna, Northern Province, Sri Lanka; 9 Institute of Applied Health Research, University of Birmingham, Birmingham, Birmingham, UK; 10 Liverpool Centre for Cardiovascular Science, University of Liverpool, Liverpool, Merseyside, UK; 11 Department of Applied Health Sciences, University of Birmingham College of Medical and Dental Sciences, Birmingham, Birmingham, UK; 12 University of Sao Paulo, São Paulo, SP, Brazil; 13 Department of Community and Family Medicine, University of Jaffna Faculty of Medicine, Jaffna, Northern, Sri Lanka; 14 University of Birmingham, Birmingham, West Midlands, UK; 15 Department of Public Health, Epidemiology and Biostatistics, University of Birmingham College of Medical and Dental Sciences, Birmingham, West Midlands, UK

**Keywords:** Global Health, Health education and promotion, Health services research, Health systems, Cardiovascular disease

## Abstract

**Introduction:**

Information continuity and self-care are important for optimal management of atrial fibrillation (AF) to reduce complications (eg, stroke) and improve prognosis and patient satisfaction. This can be achieved through handover of information from healthcare professionals (HCPs) to patients.

**Methods:**

In Brazil, China and Sri Lanka, we conducted a mixed-methods study to identify cross-country differences, similarities, barriers and facilitators regarding HCP-to-patient handover on AF. Adults (≥18 years) with AF who spoke the local languages were included. Anyone with hearing or cognitive impairment was excluded. A questionnaire was administered and focus group discussions (FGDs) conducted. χ^2^ tests identified differences within and between countries on use of patient-held health records (PHRs); a content analysis identified perspectives and experiences of HCP-to-patient handover. Data were then triangulated using a convergence model to compare and contrast quantitative and qualitative findings to identify barriers and facilitators for improving HCP-to-patient handover.

**Results:**

716 participants completed the questionnaire and 13 FGDs were conducted. People with AF receive a range of information on living with AF and AF management, though information given varies between countries. All three countries had PHRs and most patients said they were important and were used by doctors; however, PHRs were inconsistently given to patients and updated by doctors. Although patients valued the information provided, PHRs were not often used for their dual purpose (self-care and information continuity), and often, patients used external sources for seeking additional information which was difficult for disadvantaged patients, particularly in China.

**Conclusion:**

Our findings highlight inefficiencies of HCP-to-patient handover for AF that have implications on healthcare and patient safety in low- and middle-income countries (LMICs). A global standard is needed to describe what information PHRs should include. Additionally, HCPs and patients should be informed on how to optimise PHRs and handover practices to improve self-care, support, prognosis and healthcare resilience of AF care in LMICs.

**WHAT IS ALREADY KNOWN ON THIS TOPIC**

- Information continuity is the transfer of patient health information across the healthcare system and is essential for patient safety and optimal management of chronic conditions such as atrial fibrillation (AF), a major risk factor for stroke. Patient-held health records (PHRs) are one way to ensure information continuity through healthcare professional (HCP)-to-patient handover and they also play a key role in self-care and self-management of AF, which is critical for improving prognosis and reducing AF-related complications. For PHRs to be an effective form of HCP-to-patient handover, HCPs must consistently check and update information on them, and patients must bring them to every healthcare visit and refer to them at home for self-care/management. However, evidence is lacking on current practices of HCP-to-patient handover for AF in low- and middle-income countries (LMICs), where AF burden and AF-related strokes are common and increasing.

**WHAT THIS STUDY ADDS**

- The use and perception of PHRs differ across three diverse countries, in part due to context-specific factors; however, many similar facilitators and barriers were identified. Most patients with AF thought PHRs were important; however, many did not appreciate the dual purpose of PHRs (i.e., information continuity and self-care). While patients are universally provided information on living with AF and AF management (written and/or verbal), this information varied between countries and often led to informal information seeking behaviours, indicating the importance of developing a comprehensive global information standard, tailored for resource-limited settings, for HCP-to-patient handover to optimise self-care, continuity of care and prognosis for AF.

**HOW THIS STUDY MIGHT AFFECT RESEARCH, PRACTICE OR POLICY**

- Our findings from three diverse, structurally and culturally different LMICs (Brazil, China and Sri Lanka) highlight implications for healthcare and patient safety relevant to other LMICs. These results should guide national and international recommendations on how HCP-to-patient handover can be improved to optimise self-care, support, prognosis and healthcare resilience for AF care in LMICs. This may include: policy and training for HCPs on the importance and methods of communication to encourage doctors to discuss management in lay terms and ideally provide take-home information to patients, on how they can self-manage AF to improve clinical outcomes; policy and practice on the use of and minimum standard of information to include in PHRs to improve the quality and effectiveness of AF care and educational initiatives for ensuring HCPs and patients understand the dual purpose of PHRs and their importance irrespective of having robust electronic healthcare records.

## Introduction

 Stroke led to 6.6 million deaths in 2019, 86% of which occurred in low- and middle-income countries (LMICs).^1^ A major risk factor for stroke is atrial fibrillation (AF), which is the most common heart arrhythmia. Oral anticoagulants (OACs) such as warfarin can reduce the risk of stroke by 64% and all-cause mortality by 26%, compared with control or placebo^2^; however, close monitoring of and often regular adjustments to warfarin are required to reduce the risk of haemorrhage and thrombosis.^3^ Many medications and clinical procedures are also contraindicated for patients on warfarin due to severe and sometimes fatal interactions and adverse effects.^4^ Although non-vitamin K OACs (NOACs) require less follow-up than warfarin while providing similar efficacy,^5^ they have limited availability in LMICs.^6^

It is critical for healthcare professionals (HCPs) across the healthcare system to know if their patients have AF and the details of their treatment plan, particularly when on warfarin; however, this is dependent on information continuity where appropriate patient information is consistently recorded, received and used among HCPs within and between healthcare facilities. Information continuity is key for patient safety.^7^ In addition to information continuity, patients with AF must be empowered and well-informed about how to take care of themselves and self-manage their condition outside the healthcare setting to improve prognosis. In LMICs, information continuity has been found to be suboptimal for chronic conditions.^8^ Such inefficiencies in informational continuity and lack of patient knowledge, attitudes or behaviours contribute to increased AF-related complications, including stroke.^6 9 10^

The global increase in pluralistic healthcare systems complicates information continuity for AF and other non-communicable diseases (NCDs), with evidence suggesting that people often use a mixture of public, private and traditional health services to receive care; this is especially true in LMICs.^11 12^ Lack of service integration and electronic records in LMICs also create obstacles for achieving information continuity.^13^ In such healthcare systems, information continuity often relies on the verbal or written exchange of information between HCPs and patients, known as HCP-to-patient handover. This enables the patient to transfer information to HCPs at their subsequent visit about their condition(s) and medication(s) and is also important for improving patient knowledge, attitudes and behaviour towards optimal self-care. HCP-to-patient handover may include information regarding their condition, risk and signs of complications, current medication and doses, treatment options, instructions on medication use, potential medication side effects and/or how they can prevent complications and improve prognosis.

For AF care, information for improving patient knowledge on bleeding risks, compliance with medication and daily activities they should or should not do to reduce complications is vital to improve prognosis.^14^ Written communication through use of patient-held health records (PHRs) is one way to achieve HCP-to-patient handover, enabling the patient to take PHRs home and refer to for better understanding of their condition and at-home care. Patients can also take PHRs to the various HCPs they may visit (for AF or other conditions) to aid HCPs in making safe and effective decisions on treatment and care.

Evidence suggests that the availability and use of PHRs for NCDs are limited in LMICs,^15^ and evidence is lacking in general on practices of HCP-to-patient handover. No study to date has explored patients’ perspectives on AF-related HCP-to-patient handover. Therefore, we conducted a mixed-methods study aimed to identify cross-country differences, similarities, barriers and facilitators regarding HCP-to-patient handover of AF-related information in Brazil, China and Sri Lanka.

## Methods

### Study design

This convergent mixed-methods study formed a component of a larger study that aimed to identify the pathways of care for people with AF in Brazil, China and Sri Lanka.^16^ The quantitative component comprised a questionnaire regarding use of PHRs and the qualitative component comprised focus group discussions (FGDs) to explore patients’ experiences and opinions on any HCP-to-patient handover (written or verbal).

### Study setting and participants

The study sites within each country are described in detail elsewhere.^9 16^ In brief, participants were recruited from 11 primary care facilities and two cardiology outpatient departments (secondary care facilities) within the Butantan area of São Paulo, Brazil; cardiology outpatient departments from two tertiary care facilities (Beijing and Shanxi) and one secondary hospital (Shanxi) in China and the cardiology and medical outpatient department and accident and emergency (A&E) department from the only tertiary care facility in Jaffna, Sri Lanka. Patients from these facilities are largely representative of the local AF population retained in care. Patients with a confirmed diagnosis of AF or an arrhythmia likely to be AF, aged 18 years or older and who spoke the local languages, were eligible for inclusion. Patients were excluded if they had hearing or cognitive impairment or did not live in São Paulo, Beijing, Shanxi or the Northern Province of Sri Lanka.

### Participant recruitment

Electronic records were used to identify eligible patients in Brazil and China. All eligible patients were invited to participate in the quantitative component of the study, and a subsample of patients were also invited to participate in the qualitative component of the study. Participants in Brazil were purposively sampled^17^ for the qualitative component based on sex, age and sociodemographic status. In China, participants were purposively sampled^17^ and organised into FGDs based on age and education level. In Sri Lanka, trained research staff identified eligible patients consecutively visiting the outpatient clinics, approached them in person and invited them to participate in the quantitative component; from those that took part, participants for the qualitative component were purposively recruited^17^ based on age, sex and sociodemographic status. To reach saturation from the FGDs,^18^ we aimed to conduct at least three FGDs in each country.

### Data collection

Quantitative data collection took place first, followed by the qualitative data collection. All data were collected between June 2019 and November 2020 in Brazil, between July 2019 and July 2021 in China and between October 2020 and June 2021 in Sri Lanka.

#### Quantitative component

The questionnaire was adapted from studies conducted in Mongolia and India; it was developed in English by the research team located at the People’s Liberation Army (PLA) General Hospital in China and the Universities of São Paulo, Jaffna, Birmingham and Liverpool. The questionnaire was translated into Portuguese, Mandarin and Tamil, then back translated into English to check for accuracy. Piloting of the questionnaire among 10 patients in Brazil and four in Sri Lanka resulted in no major changes made to the questionnaire. Data were collected over the phone or face-to-face in Brazil and China and face-to-face in Sri Lanka. Further details regarding data collection are available in a separate publication.^16^

#### Qualitative component

The topic guide was developed in English by the research teams in Brazil, China and Sri Lanka and qualitative experts in the UK (SG and SMH). They were the same across all three countries and translated into Portuguese, Tamil and Mandarin, then back translated into English to check for accuracy. The topic guides were piloted in each setting, but no major changes resulted. In Brazil, two trained nurses took notes of the FGDs alongside a clinician and professor of public health (ACG) and anthropologist (EP) who led the discussions; participants sat at a round table in a private room within the Hospital Universitário of the University of São Paulo. FGDs in China were facilitated by attending cardiologists (HW, HZ, JZ, MF and XL) and conducted virtually or in person in a private consultation room or patient education area at the PLA General Hospital in Beijing or in Shanxi at Shanxi Bethune Hospital or Xiaodian District Wucheng Community Health Service Centre. In Sri Lanka, FGDs were facilitated by senior lecturers (BK and SS) and three early career researchers (SAV, PU and SK) and conducted in a staff meeting room within the Jaffna Teaching Hospital. All FGD facilitators were trained in qualitative methods and had no prior relationship with participants.

### Data analysis

#### Quantitative component

The sample size calculation was based on the aims of the affiliated study for identifying AF care pathways,^16^ where a minimum sample size of 236 was required for each country. Descriptive statistics were used to present baseline demographics, where mean and SD are presented for continuous variables and frequency and percentages are presented for categorical variables. Questions of interest were categorised based on HCP and patient behaviour regarding handover; these questions were investigated using a χ^2^ test to identify differences in response data between countries and between subgroups within each country. The following subgroups were investigated: age (<70 years, 70+years), sex (male, female), education (no education, primary education, secondary or higher education), employment (employed, unemployed, retired), marital status (single, married), AF duration (<5 years, 5–10 years, >10 years) and OAC use (warfarin, NOACs, no OACs). To increase power, anyone divorced or widowed was categorised as single.

#### Qualitative component

All interviews were audio recorded and transcribed verbatim. Transcripts were first written in the local languages of Portuguese, Mandarin and Tamil for Brazil, China and Sri Lanka, respectively. All transcripts and notes were then translated into English. A content analysis^19^ was conducted separately for each country; all transcripts were read line-by-line in full, coding any data related to handover where handover was defined as any exchange of information between HCPs and patients, either written or verbally.

TEG analysed the data from all three countries (starting with transcripts from Brazil, then Sri Lanka, then China) and themes were developed through iterations of coding. TEG consulted with researchers in Brazil (ACG and EP), China (HW) and Sri Lanka (SAV) throughout the analysis process, all of whom were present during the respective FGDs. TEG, a research fellow and PhD candidate in global health, resides in the UK and is experienced in qualitative methods; she was not present for any FGDs and has no relationship with the participants. The analysis was supervised by qualitative experts (SG and SMH) from the University of Birmingham, UK.

#### Mixed methods analysis

Using our former perspective of health systems, HCP and patient factors,^20 21^ quantitative and qualitative data were triangulated to identify barriers and facilitators for improving HCP-to-patient handover for AF from the patients’ perspective. We used a convergence model of triangulation,^22^ first analysing the quantitative and qualitative data separately, then comparing and contrasting the findings from each.

### Patient and public involvement

Two people with AF aided in the conceptualisation, planning and delivery of this research. Each country had people with AF codevelop plans for local dissemination of results.

## Results

### Participant demographics

Quantitative data were collected from 267 AF patients in Brazil, 298 in China and 151 in Sri Lanka, representing 64%, 77% and 100% of all eligible participants identified, respectively. Demographics are presented in table 1 and described in detail elsewhere.^16^ None of the patients invited to participate in the FGDs declined. Three FGDs were conducted each in Brazil (n=17) and Sri Lanka (n=25) and seven were conducted in China (n=33). Overall, participants were well balanced in terms of age groups across the FGDs in Brazil and Sri Lanka; by design, China FGDs differed by age groups (table 1). Females represented 70% of participants in Sri Lanka, 62% in China and 49% in Brazil. Most FGD participants were married.

**Table 1:** Participant demographics from the quantitative component of the study

	Brazil		China		Sri Lanka	
	Quant (n=267)	Qual (n=17)	Quant (n=298)	Qual (n=33)	Quant (n=151)	Qual (n=25)
Age						
Median years (IQR)	71 (61–77)	58 (55–67)	66 (58–74)	66 (47–83)	57 (49–67)	55 (32–71)
<50 years	16 (6.0)	1	34 (11.4)	1	43 (28.5)	11
51–69 years	106 (39.7)	14	159 (53.4)	19	84 (55.6)	9
≥70 years	145 (54.3)	2	105 (35.2)	13	24 (15.9)	5
Sex						
Female	131 (49.1)	8	113 (37.9)	9	106 (70.2)	18
Male	136 (50.9)	9	185 (62.1)	24	45 (29.8)	7
Marital status						
Single	26 (9.7)	1	3 (1.0)	0	19 (12.6)	2
Married or living with partner	160 (59.9)	13	280 (94.0)	31	94 (62.3)	14
Divorced	24 (9.0)	2	2 (0.7)	1	7 (4.6)	1
Widowed	51 (19.1)	1	5 (1.7)	1	31 (20.5)	8
Missing/unknown	6 (2.3)	0	8 (2.7)	0	0 (0)	0
Ethnicity*						
White	134 (50.2)	9	–	–	–	–
Black	31 (11.6)	2	–	–	–	–
Mixed	76 (28.5)	6	–	–	–	–
Han	–	–	298 (100)	33	–	–
Sri Lankan Tamil	–	–	–	–	146 (96.7)	24
Other	6 (2.3)	0	0 (0)	0	5 (3.3)	1
Missing/unknown	20 (7.5)	0	0 (0)	0	0 (0)	0
Education						
Did not complete primary school	86 (32.2)	1	18 (6.0)	2	22 (14.6)	8
Completed primary school	91 (34.1)	3	27 (9.1)	3	40 (26.5)	12
Completed secondary education	57 (21.4)	11	149 (50.0)	12	86 (57.0)	3
Holds undergraduate degree	20 (7.5)	1	98 (32.9)	14	3 (2.0)	2
Holds postgraduate degree	3 (1.1)	0	6 (2.0)	2	0 (0)	0
Missing/unknown	10 (3.8)	1	0 (0)	0	0 (0)	0
Employment status						
Employed	44 (16.5)	7	63 (21.1)	7	27 (17.9)	6
Retired	182 (68.2)	4	211 (70.8)	24	12 (8.0)	12
Housewife	16 (6.0)	6	5 (1.7)	2	89 (58.9)	4
Student	1 (0.4)	0	2 (0.7)	0	0 (0)	2
Unable to work	20 (7.5)	0	9 (3.0)	0	19 (12.6)	1
Cannot find work	4 (1.5)	0	1 (0.3)	0	1 (0.7)	0
Does not want to work	0 (0)	0	2 (0.7)	0	0 (0)	0
Missing	0 (0)	0	0 (0)	0	3 (2.0)	0
Time since AF diagnosis						
Median months (IQR)	96 (36–180)	120 (42–228)	24 (5–62)	31 (4–85)	40 (20–70)	46 (29–71)
<5 years	90 (33.7)	6	203 (68.1)	19	105 (69.5)	13
5–10 years	69 (25.8)	2	37 (12.4)	9	30 (19.9)	7
11+years	81 (30.3)	9	34 (11.4)	5	14 (9.3)	3
Unknown	27 (10.1)	0	24 (8.1)	0	2 (1.3)	2
Anticoagulation						
Taking warfarin	228 (85.4)	17	61 (20.5)	2	150 (99.3)	25
Taking NOACs	16 (6.0)	0	139 (46.6)	13	0 (0)	0
Not taking anticoagulant	23 (8.6)	0	98 (32.9)	18	1 (0.7)	0

* Data are shown for ethnicity groups relevant to each country. A dash is presented where the ethnicity group is not relevant.

AF, atrial fibrillation; NOAC, non-vitamin K oral anticoagulant; Qual, qualitative; Quant, quantitative.

### Mixed-methods results

**Figure 1:**
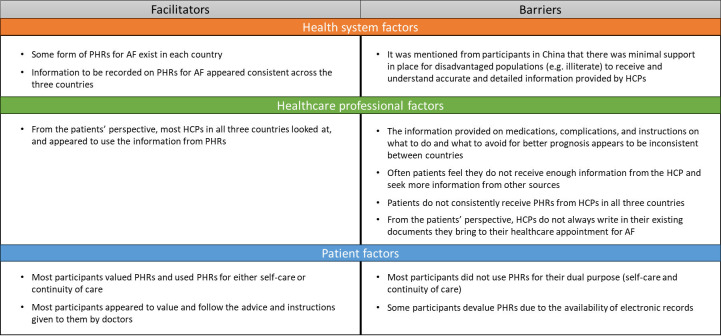
Facilitators and barriers for improving HCP-to-patient handover for AF. AF, atrial fibrillation; HCP, healthcare professional; PHR, patient-held health record.

The complete and separate analyses for the quantitative and qualitative data are presented in the online supplemental materials. After integrating the quantitative and qualitative results, we identified several barriers and facilitators for optimising HCP-to-patient handover for AF related to healthcare system, HCP and patient factors (figure 1).

#### Health system factors

Two *facilitators* were identified as health system factors (table 2):

**Table 2:** Supporting data for healthcare system barriers and facilitators

		Mixed-methods findings	Supportive qualitative data	Supportive quantitative data
Healthcare system factors	**Facilitators**	PHRs are available for AF in each country.	*“There are papers they give, but the exam stays there with them. What they give us is the [INR results*]*, we do it and take the document.”* (Brazil, FGD 3, P5) *“The doctor gave me a paper medical record, examination results and prescription.”* (China, FGD 1, P8) *“In this [clinic] book I had all my things from the cardiologist.”* (Sri Lanka, FGD 2, P3)	Only 3% (8/267) of participants from Brazil, 6% (19/298) from China and 0% (0/151) from Sri Lanka said they were not given a note or document that lists their medicines. Only 5% (16/298) of participants from China and 10% (15/151) from Sri Lanka said they were not given a note or document to bring to their next appointment. Although the proportion was higher in Brazil, it was still less than half (46%; 124/267).
		Information on AF PHRs appeared consistent across the three countries.	*“What they give us is the [INR results], we do it and take the document.”* (Brazil, FGD 3, P5) *“At most, there are prescriptions. There are also some test sheets, including some indicators like blood lipids. But very few are written by hand with few words.”* (China, FGD 3, P1) *“All the test and treatment which were taken are mentioned in this [clinic] book and our phone numbers also present here.”* (Sri Lanka, FGD 1, P8)	NA
	**Barriers**	Minimal support is in place in China for disadvantaged populations to receive and understand information on AF.	*“Due to my low educational background, I can’t use the Internet or books to look up relevant information.” (China, FGD 5, P1*) *“Because I have to take care of my wife every day, I don’t have the time and energy to find information about atrial fibrillation by myself.” (China, FGD 5, P2*) *“I want to communicate with people who is knowledgeable about AF but I don’t have such a good friend.” (China, FGD 2, P3*)	NA

AF, atrial fibrillation; FGD, focus group discussion; INR, International normalised ratio; P, patient; PHR, patient-held health record.

1. Some form of PHRs for AF exist in each country. All participants from Brazil, China and Sri Lanka spoke about and knew of documents given to them from their doctor to take home with information regarding their condition, medication and/or test results. This was evidenced by the small proportion of people that said they were not given a note or document that lists their medicines (3%, 19% and 0% from Brazil, China and Sri Lanka, respectively) or a note or document to bring to their next AF appointment (47%, 5% and 10% from Brazil, China and Sri Lanka, respectively)
2. Information to be recorded on PHRs for AF appeared consistent across the three countries. Participants from all three countries said that international normalised ratio (INR) results, prescriptions and dose are to be recorded on their PHRs. Sri Lankan participants said their PHR (ie, clinic book) also has their address and phone number included.

One *barrier* for optimising HCP-to-patient handover for AF was found regarding health system factors (table 2):

It was mentioned from participants in China that there was minimal support in place for disadvantaged populations (eg, illiterate) to receive and understand accurate and detailed information provided by HCPs. Participants from China with a higher education status recalled more detailed information that doctors gave them about AF management and complications, and expressed a greater understanding of the options available to manage/treat their AF. However, it was mentioned by a few participants with a lower education status that they lack access to additional sources of information. For instance, one participant was illiterate, and another did not have time due to caring responsibilities, indicating that socioeconomic status may impact a patient’s ability to understand information and seek information outside a hospital setting.

### HCP factors

One *facilitator* was found for HCP factors (table 3):

**Table 3:** Supporting data for healthcare professionals’ barriers and facilitators

		Mixed-methods findings	Supportive qualitative data	Supportive quantitative data
Healthcare professional factors	**Facilitators**	From the patients’ perspective, most HCPs in all three countries looked at and used existing PHRs.	*“But my God, they want to know everything, right people? How was the story and you have to have the documents to prove it.”* (Brazil, FGD 2, P5) *“I have hard copies of medical records, and I keep them well. [The doctor] will compare the results of the previous examination with the present one.”* (China, FGD 7, P1) *“We receive medication by showing this clinic book and informing that we are having this disease.”* (Sri Lanka, FGD 3, P8)	Of the participants that brought a document with them to their most recent AF-related healthcare appointment, 92% (108/117) from Brazil, 95% (178/187) from China and 90% (120/134) from Sri Lanka said the doctor used the document they brought (χ^2^ p value: 0.158). This differed by age in Brazil: More participants <70 years old said yes compared with those ≥70 years old (97% vs 87%, p value=0.048).
	**Barriers**	Information provided on medications, complications and how to improve prognosis appears to be inconsistent between countries.	“*I avoid the green leaf more, but I like it, but I avoid it… I know what is needed, the doctor guided me.”* (Brazil, FGD 2, P4) *“I don't remember [how long I have to take warfarin], I don’t think they informed me, I think it’s forever.”* (Brazil, FGD 3, P4) *“Doctors have introduced to me what is atrial fibrillation, the incidence of atrial fibrillation, harm and related treatment. Treatment includes heart rate control, anticoagulation, thrombosis prevention and radiofrequency ablation.”* (China, FGD 1, P10) *“We were informed that it will be difficult if get any wound, bleeding won’t stop, in order to control bleeding, seek hospital immediately, need to put ice.”* (Sri Lanka, FGD 1, P4)	When participants were asked whether the doctor explained how to take care of yourself: 73% (187/256) from Brazil, 80% (210/264) from China and 72% (108/151) from Sri Lanka said, ‘yes, very well’. This differed based on warfarin use in China where participants on warfarin were more likely to say this compared with participants on NOACs or OACs (95% vs 72% vs 82% respectively, p value=0.002). 18% (47/256) from Brazil, 19% (49/264) from China and 27% (40/151) from Sri Lanka said, ‘yes, but not very well’. 9% (22/256) from Brazil, 2% (5/264) from China and 2% (3/151) from Sri Lanka said, ‘no’. χ^2^ p value <0.001
		Patients in Brazil and China do not receive enough information from HCPs and seek information from other sources.	*“I searched Google [for more information*]. *Doctor Google. I put how it happened, what it is, what provokes it, what causes it.”* (Brazil, FGD 1, P1) *“I didn’t know there was a [risk of] blood clot. My son once told me that AF can cause blood clots.”* (China, FGD 3, P2) *“I looked up the information related to medicine on my mobile phone. I think I should be cautious; maybe Traditional Chinese medicine will be better.”* (China, FGD 6, P3)	When participants were asked whether the doctor explained how to take care of yourself: 73% (187/256) from Brazil, 80% (210/264) from China and 72% (108/151) from Sri Lanka said, ‘yes, very well’. This differed based on warfarin use in China where participants on warfarin were more likely to say this compared with participants on NOACs or OACs (95% vs 72% vs 82%, respectively, p value=0.002) 18% (47/256) from Brazil, 19% (49/264) from China and 27% (40/151) from Sri Lanka said, ‘yes, but not very well’. 9% (22/256) from Brazil, 2% (5/264) from China and 2% (3/151) from Sri Lanka said ‘no’. χ^2^ p value <0.001
		Patients do not consistently receive PHRs from HCPs in all three countries.	*“[The doctor] doesn’t give me a report, but he gives me a request that says ‘Take [the medication] so you don’t forget’.”* (Brazil, FGD 2, P1) *“Doctors sometimes give me paper medical records, sometimes they don’t. I want a paper medical record. It’s useful. Paper cases can help me to observe the changes of my condition.”* (China, FGD 1, P4)	When participants were asked if they brought a document given to them previously, 46% (124/267) from Brazil, 5% (16/298) from China and 10% (15/151) from Sri Lanka said, ‘no, I had not been given anything to bring’ (χ^2^ p value <0.001).
		From the patients’ perspective, HCPs do not always write in their existing documents they bring.	*“Even if I take [documents to my next appointment], the doctor will not look at it patiently. There are so many people waiting to see a doctor. The doctor ended up talking with a few words in several minutes.”* (China, FGD 3, P1)	When participants were asked whether the doctor wrote in their documents, 49% (57/117) from Brazil, 96% (172/180) from China, 52% (70/134) from Sri Lanka said ‘yes’ (χ^2^ p value <0.001).

AF, atrial fibrillation; FGD, focus group discussion; HCP, healthcare professional; P, patient; PHR, patient-held health record.

From the patients’ perspective, most HCPs in all three countries looked at and appeared to use the information from PHRs. Of those that brought their documents to their most recent healthcare appointment for AF, ≥90% of participants from Brazil, China and Sri Lanka said the doctor used their documents. One of the reasons that participants in Brazil said they bring their documents to their healthcare appointments was because the doctors always ask to see their PHRs. Participants in Sri Lanka said that PHRs are sometimes necessary to be seen by the HCP or have their prescriptions refilled. While younger participants in Brazil (<70 years) were more likely to bring a document compared with older participants (70+years), the proportion was still high for both (97% vs 87%, p value=0.048).

Four *barriers* were found to be HCP factors (table 3):

1. The information provided on medications, complications and instructions on what to do and what to avoid for better prognosis appears to be inconsistent between countries. Advice on lifestyle behaviours was provided to participants in China including the impacts of diet, exercise, smoking and drinking alcohol; however, participants in Brazil said they were only provided advice on diet and nutrition (eg, avoid green leafy vegetables rich in vitamin K due to warfarin use), and in Sri Lanka, advice on diet/nutrition as well as exercise and social events/interactions (eg, avoid loud noises or vibrations) was provided. Participants from Sri Lanka were told about and understood the importance of adhering to warfarin, when and how to take it and the purpose of taking it lifelong; they were also told about the signs of complications to be aware of and what to do when they occur. While some participants from Brazil and China were told detailed information regarding warfarin, this was not true for all; some participants from Brazil, for instance, admitted to not knowing exactly what AF was and were therefore unsure how warfarin affected it. Regarding complications, Brazilian participants did not appear to have been told this information; however, participants from China were told the types of complications and events that AF could cause, the aetiology of such events and how they can be prevented, such as for thrombosis and heart failure. Participants from China were also informed about additional medical procedures that could be used including ablation and cardioversion. Indeed, more participants from China (80%) said the doctor explained very well how to take care of themselves compared with participants from Brazil and Sri Lanka (73% and 72%, respectively), and those on warfarin in China were more likely to say this compared with participants on NOACs or OACs (95% vs 72% vs 82%, respectively).
2. Often patients feel they do not receive enough information from the HCP and seek more information from other sources. Nearly a fifth of participants said the doctor did not explain how to take care of themselves very well in Brazil (18%) and China (19%) and a quarter of participants said this in Sri Lanka (27%). Participants from Brazil and China said they seek additional information from a range of sources including the internet, family and friends (including others with AF) and other doctors and facilities (including traditional medicine hospitals).
3. Patients do not consistently receive PHRs from HCPs in all three countries. Nearly half of participants from Brazil (124/267, 46%) said they had not been given a PHR to bring to their most recent appointment. 5% (16/298) of participants in China and 10% (15/151) in Sri Lanka said this. Participants from Brazil and China spoke about this in the FGDs, stating that sometimes they get documents and sometimes they do not.
4. From the patients’ perspective, HCPs do not always write in their existing documents they bring to their healthcare appointment for AF. While most participants in China said doctors write in their documents (172/180; 96%), half of participants from Brazil (60/117; 51%) and Sri Lanka (64/134; 48%) said “no/don’t know”. However, only participants that brought a document with them to their most recent healthcare visit were asked this question. Participants from China admitted that they often do not bring their PHRs with them, in part because doctors do not use or write in them. Indeed, some FGD participants from China who do bring their PHRs to appointments said their PHRs are incomplete and missing information regarding prescriptions and blood tests.

### Patient factors

Two *facilitators* for optimising HCP-to-patient AF handover were found to be patient factors (table 4):

**Table 4:** Supporting data for patient barriers and facilitators

		Mixed-methods findings	Supportive qualitative data	Supportive quantitative data
Patient factors	**Facilitators**	Most participants value PHRs and use PHRs for either self-care or continuity of care.	*“I do think it’s important to have because every place we go they ask us to tell our story, but it’s no use just talking, they must see everything that happened to us.”* (Brazil, FGD 2, P5) *“It is necessary for a doctor to give a paper medical record. There are too many patients; it is difficult for the doctors to remember everyone’s condition.”* (China, FGD 1, P2) *“While we were displaced due to war, we carried this copy carefully as same as money and jewels.”* (Sri Lanka, FGD 1, P4)	When participants were asked if they thought written documents were important, 74% (196/266) from Brazil, 95% (266/280) from China and 90% (136/151) from Sri Lanka said ‘yes’ (χ^2^ p value <0.001). This differed based on sex and employment status in China: More males said yes compared with females (98% vs 91%, p value=0.010) More employed participants said yes compared with unemployed and retired participants (100% vs 83% vs 94% respectively, p value=0.016) When participants were asked if they use their documents at home, 97% (256/265) from Brazil, 94% (272/288) from China and 74% (111/151) from Sri Lanka said ‘yes’ (χ^2^ value <0.001). This differed by sex in China: More females said yes compared with males (98% vs 92%, p value =0.030)
		Most participants in all three countries appeared to value and follow the advice and instructions given to them by HCPs.	*“I don’t eat anything green because of Warfarin … not like kale, stuff like that. They say it changes, right? Mine changes if I eat.”* (Brazil, FGD 1, P5) *“All I know is that I should take the medicine as prescribed by my doctor. Also, I should pay attention to lifestyle.”* (China, FGD 4, P1) *“Loud vibration is also not good, I ask my family if a funeral happened in my family let me take somewhere to avoid the loud sound and vibration.”* (Sri Lanka, FGD 1, P4)	When participants were asked if they use their documents at home, 97% (256/265) from Brazil, 94% (272/288) from China and 74% (111/151) from Sri Lanka said ‘yes’ (χ^2^ p value <0.001). This differed by sex in China: More females said yes compared with males (98% vs 92%, p value =0.030)
	**Barriers**	Many participants did not use PHRs for their dual purpose (self-care and continuity of care).	NA	When participants were asked if they brought a document given to them previously, 44% (177/264) from Brazil, 65% (193/298) from China and 89% (134/151) from Sri Lanka said ‘yes’ (χ^2^ p value <0.001). This differed by age in Brazil: More participants <70 years old said yes compared with those ≥70 years old (52% vs 38%, p value=0.020) When participants were asked if they use their documents at home, 97% (256/265) from Brazil, 94% (272/288) from China and 74% (111/151) from Sri Lanka said ‘yes’ (χ^2^ p value <0.001). This differed by sex in China: More females said yes compared with males (98% vs 92%, p value=0.030)
		Some participants from Brazil and China devalue PHRs, often due to the availability of electronic records.	*“I know that through the computer I can see all the [hospital] results. I access it through the internet and through the Hospital das Clínicas, I access it and get it there, endoscopy, blood test, I get everything.”* (Brazil, FGD 2, P4) *“I don't think it’s necessary for doctors to give paper cases because computers keep records.”* (China, FGD 1, P7) *“They have given [documents] to me. But I lost them long ago.”* (China, FGD 4, P2)	NA

FGD, focus group discussion; HCP, healthcare professional; P, patient; PHR, patient-held health record.

1. Most participants valued PHRs and used PHRs for either self-care or continuity of care. Most participants in China (266/280; 95%) and Sri Lanka (136/151; 90%) said that written documents for them to take home are important; however, only 74% of participants in Brazil said this (196/266). In China, males were more likely to say this compared with females, though the proportion was high for both (98% and 91%, respectively). Also in China, all employed participants said they were important (100%) compared with 83% of unemployed and 94% of retired participants. Participants from Sri Lanka emphasised how important the clinic book was to them, one participant stating they were the same as money and jewels. They stated the extent they go to protect it (eg, laminating it or making a copy) and how long they have kept the PHRs (often decades); many mentioned the reason for this was the recent civil war in Sri Lanka that caused extensive displacement and difficulties with accessing healthcare. Several participants from China said PHRs are helpful and important for them to keep track of any changes to their health and/or for doctors because they cannot remember details for all patients.
2. Most participants appeared to value and follow the advice and instructions given to them by doctors. Participants from all three countries appeared grateful for the information and advice given on daily living. Many discussed how they made great efforts to follow the instructions, including avoiding funerals (for Sri Lankans) and completely avoiding foods rich in vitamin K (eg, leafy green vegetables). This again was exemplified by the high proportion of people that used their documents at home.

Two *barriers* were identified as patient factors (table 4):

1. Most participants did not use PHRs for their dual purpose (self-care and continuity of care). Nearly all participants in Brazil (97%) and China (94%) said they refer to PHRs given by their doctors about AF care while at home (ie, used them for self-care); however, less than half of participants from Brazil (117/264; 44%) brought PHRs to their most recent appointment and only 65% (193/298) of participants from China did (ie, used them for continuity of care). Conversely, more participants from Sri Lanka said they brought their PHRs to their healthcare appointment (134/151; 89%) (ie, used them for continuity of care) compared with the proportion that said they refer to PHRs at home (111/151; 74%) (ie, used them for self-care). There were a couple of differences found across subgroups in Brazil and China: younger participants (<70 years) in Brazil were more likely to bring their documents to their healthcare appointment, and in China, females were more likely to use their documents at home.
2. Some participants devalue PHRs due to the availability of electronic records. Some participants from Brazil and China did not see the importance of PHRs in part because their health records were available electronically. Those from Brazil said they can access these records from home by logging into a patient portal online or through a mobile application. Many participants from China said they do not bring documents to their healthcare appointments because their information is already available to the HCPs when they arrive, and one participant said they lost their documents long ago.

## Discussion

We report findings from a mixed-methods study on the cross-country differences, similarities, barriers and facilitators regarding HCP-to-patient handover of AF-related information from three diverse LMICs. Our results showed that people with AF receive a range of information on living with AF and AF management. However, information given appears to vary between countries. All three countries had some form of PHRs with consistent information recorded, reportedly used by most doctors and thought to be important by most participants. However, the dual purpose of PHRs was not understood by most patients. Importantly, patients understood the seriousness of AF and tried to follow instructions provided by HCPs; however often patients had to resort to external sources for information which was difficult for disadvantaged patients or patients with lower socioeconomic status. These facilitators and barriers were categorised as health system, healthcare provider and patient related factors for which the most barriers were healthcare provider factors.

The variation across countries on the information provided about living with AF could be because they were not told all the necessary information, they forgot the information or they did not think it was important to mention. There is evidence that patients (including in high-income countries) do not always report events accurately from doctor consultations due to these reasons.^23^ Promoting healthy lifestyle behaviours is a pillar of the AF Better Care (ABC) pathway^24^ and it is recommended for doctors to discuss (in a way that patients can understand and remember) weight loss, regular physical activity, alcohol reduction and smoking cessation with AF patients for a better prognosis.^25^,^28^ Adherence to the ABC pathway has been shown to reduce AF complications such as stroke and the development of comorbidities^29 30^ and has been shown to be a cost-effective strategy.^31^ National guidelines, policy and training for HCPs on the importance and methods of communication could help encourage doctors to discuss in lay terms and ideally provide written information to patients, on which lifestyle behaviours they could change to improve clinical outcomes of AF.^25^,^28^ However, patients having knowledge does not often translate to behaviour change; existing evidence on various methods to elicit lifestyle behaviour change, successfully and sustainably through more complex programmes involving non-monetary incentives and changes in social norms through larger fiscal policies and community level campaigns should be adapted and tested in different cultural settings.^32^,^34^

Given patients often seek care from different sources, a single universal PHR is a useful tool for achieving information continuity of care and patient self-care, even in the presence of electronic healthcare records.^35^ PHRs can aid in improving clinical outcomes, patient satisfaction and patient safety,^15^ especially given many patients with AF have multiple other NCDs that require visits to different doctors. To optimise PHRs, HCPs must consistently enter patients’ clinical details on PHRs, patients must bring the PHRs to every healthcare visit, HCPs must refer to PHRs before making clinical decisions and PHRs must include components useful for self-management and continuity of care.^20 35^ However, many participants said doctors did not write in their documents and many participants from China and Brazil do not bring a PHR to their healthcare appointments. This could be in part because Chinese and Brazilian participants were more likely to think of PHRs as important for self-care instead of information continuity, whereas Sri Lankan participants viewed PHRs as a critical element of receiving quality and effective follow-up care through information continuity, keeping the documents with them wherever they go.

Sri Lanka is a lower-middle-income country, whereas Brazil and China are upper middle-income countries.^36^ Most of the Sri Lankan adult population has experienced adversities during the recent civil war; this is especially true for inhabitants of the Northern Province (our study setting) where the conflict was most concentrated.^37 38^ This first-hand understanding of how precarious healthcare systems can be, in addition to having less healthcare resources in general, may influence patients’ perception and use of PHRs. Patients from more developed countries or with no recent history of such adversity may fail to understand the importance of PHRs for information continuity, particularly where electronic healthcare records appear to be robust. Additionally, our previous research in Sri Lanka indicates that patients with AF have a high level of trust in HCPs,^9^ and patients’ trust in HCPs has been found to be significantly lower in East Asia and Latin America compared with South Asia.^39^ Levels of trust in HCPs is related to a complex combination of political, societal and cultural factors and may not be directly related to the individual or country income level,^40^ but may be a factor in patients understanding that HCPs can and will act in the patients’ best interest if they have appropriate information about their health status. Thus, trust in HCPs may separately impact the perception and use of PHRs. Investigating the level of patient-HCP trust and understanding the local history of healthcare delivery and political unrest may be critical to determining factors related to effective and optimal use of PHRs in LMICs in future studies.

Healthcare systems globally are vulnerable to major disruptions, as seen in the COVID-19 pandemic and increasing climate-related events (eg, floods).^41^ PHRs can be an important tool for information continuity and for patients’ ability to have clarity on their own care options; thus, PHRs must be prioritised for chronic conditions to improve patient care during and beyond major healthcare crises.^42^ To this end, PHRs should be better designed to be user-friendly and used by patients/carers for self-care at home. The lack of understanding for PHRs’ dual purpose among our diverse participants highlights the need to improve patients’ and HCPs’ attitudes and behaviours towards PHRs that ensure more complete and accurate information recorded on them and optimal use. These issues have been reported for other NCDs in LMICs, indicating a wider need for better development and use of PHRs.^15 20^ Providing standardised written information on AF could improve patients’ understanding of the condition, the options for treatment, modifiable risk factors and where they can go for further information or support, all of which were identified as knowledge gaps from our participants.

A major strength of our study is the use of a mixed-methods study design involving participants from three structurally and culturally different countries; thus, enabling an indepth understanding of HCP-to-patient handover practices and perspectives in LMICs and how these may differ across settings. From each country site, we recruited participants from all the main healthcare facilities that provide AF care; therefore, our sample is likely to be representative of people with AF retained in public care within our LMIC settings. However, in another publication, we describe in detail possible biases that may have resulted from the recruitment methods and response rate.^16^ One major limitation of our study was not providing insight from HCPs to understand from their perspective on what information they provide to patients and why. Nonetheless, and irrespective of what HCPs actually tell patients, it is important to know what patients remember, what they think is important and what they feel was missed during the handover of information, all of which have implications for patient self-care, prognosis and patient safety.

Our findings have implications for healthcare and patient safety. Variation on the availability and use of PHRs (from HCPs and patients) and what information is provided to patients with AF in Brazil, China and Sri Lanka highlight the need for establishing: (1) a global standard on using PHRs for both continuity of care and self-care and (2) an international consensus on what information should be required on PHRs to ensure self-care and continuity of care. Our findings also imply that patients from middle-income countries may require additional education on the importance and intended dual use of PHRs to warrant information continuity of care as well as self-care, while patients from lower-income countries may need education on the importance and use of such documents for self-care. These results should be used to guide national and international recommendations on how HCP-to-patient handover can be improved to optimise self-care, support, prognosis and healthcare resilience for AF care in LMICs.

## Supplementary material

10.1136/bmjgh-2024-017517 online supplemental file 1

## Data Availability

Data are available upon reasonable request.

## References

[1] Feigin VL, Stark BA, Johnson CO (2021). Global, regional, and national burden of stroke and its risk factors, 1990–2019: a systematic analysis for the Global Burden of Disease Study 2019. Lancet Neurol.

[2] Hart RG, Pearce LA, Aguilar MI (2007). Meta-analysis: antithrombotic therapy to prevent stroke in patients who have nonvalvular atrial fibrillation. Ann Intern Med.

[3] Lip GYH, Freedman B, De Caterina R (2017). Stroke prevention in atrial fibrillation: Past, present and future. Thromb Haemost.

[4] Tadros R, Shakib S (2010). Warfarin--indications, risks and drug interactions. Aust Fam Physician.

[5] Link MS, Giugliano RP, Ruff CT (2017). Stroke and Mortality Risk in Patients With Various Patterns of Atrial Fibrillation: Results From the ENGAGE AF-TIMI 48 Trial (Effective Anticoagulation With Factor Xa Next Generation in Atrial Fibrillation-Thrombolysis in Myocardial Infarction 48). Circ Arrhythm Electrophysiol.

[6] Santos IS, Goulart AC, Olmos RD (2020). Atrial fibrillation in low- and middle-income countries: a narrative review. Eur Heart J Suppl.

[7] Haggerty JL, Reid RJ, Freeman GK (2003). Continuity of care: a multidisciplinary review. BMJ.

[8] Lall D, Engel N, Devadasan N (2018). Models of care for chronic conditions in low/middle-income countries: a “best fit” framework synthesis. BMJ Glob Health.

[9] Sheron VA, Shanmugathas S, Gooden TE (2022). Healthcare provider and patient perspectives on access to and management of atrial fibrillation in the Northern Province, Sri Lanka: a rapid evaluation of barriers and facilitators to care. BMC Health Serv Res.

[10] Paschoal E, Gooden TE, Olmos RD (2022). Health care professionals’ perceptions about atrial fibrillation care in the Brazilian public primary care system: a mixed-methods study. BMC Cardiovasc Disord.

[11] Elsey H, Agyepong I, Huque R (2019). Rethinking health systems in the context of urbanisation: challenges from four rapidly urbanising low-income and middle-income countries. BMJ Glob Health.

[12] Mackintosh M, Channon A, Karan A (2016). What is the private sector? Understanding private provision in the health systems of low-income and middle-income countries. Lancet.

[13] Schwarz D, Hirschhorn LR, Kim J-H (2019). Continuity in primary care: a critical but neglected component for achieving high-quality universal health coverage. BMJ Glob Health.

[14] Snipelisky D, Kusumoto F (2013). Current strategies to minimize the bleeding risk of warfarin. J Blood Med.

[15] Joseph L, Lavis A, Greenfield S (2021). Systematic review on the use of patient-held health records in low-income and middle-income countries. BMJ Open.

[16] Gooden TE, Wang J, Carvalho Goulart A (2023). Generalisability of and lessons learned from a mixed-methods study conducted in three low- and middle-income countries to identify care pathways for atrial fibrillation. Glob Health Action.

[17] Palinkas LA, Horwitz SM, Green CA (2015). Purposeful Sampling for Qualitative Data Collection and Analysis in Mixed Method Implementation Research. Adm Policy Ment Health.

[18] Morse JM (2000). Determining Sample Size. Qual Health Res.

[19] Bengtsson M (2016). How to plan and perform a qualitative study using content analysis. *NursingPlus Open*.

[20] Humphries C, Jaganathan S, Panniyammakal J (2018). Investigating clinical handover and healthcare communication for outpatients with chronic disease in India: A mixed-methods study. PLoS One.

[21] Gooden T, Gustafsson L, Lu F (2021). Facilitating better postnatal care with women-held documents in The Gambia: a mixed-methods study. BMC Pregnancy Childbirth.

[22] Creswell JW, Plano Clark VL (2011). Designing and conducting mixed methods research.

[23] Humphries C, Jaganathan S, Panniyammakal J (2019). Patient and healthcare provider knowledge, attitudes and barriers to handover and healthcare communication during chronic disease inpatient care in India: a qualitative exploratory study. BMJ Open.

[24] Lip GYH (2017). The ABC pathway: an integrated approach to improve AF management. Nat Rev Cardiol.

[25] Chao T-F, Joung B, Takahashi Y (2022). 2021 Focused Update Consensus Guidelines of the Asia Pacific Heart Rhythm Society on Stroke Prevention in Atrial Fibrillation: Executive Summary. Thromb Haemost.

[26] Lip GYH, Banerjee A, Boriani G (2018). Antithrombotic Therapy for Atrial Fibrillation: CHEST Guideline and Expert Panel Report. Chest.

[27] Joung B, Lee JM, Lee KH (2018). 2018 Korean Guideline of Atrial Fibrillation Management. Korean Circ J.

[28] Hindricks G, Potpara T, Dagres N (2020). ESC Guidelines for the diagnosis and management of atrial fibrillation developed in collaboration with the European Association for Cardio-Thoracic Surgery (EACTS) The Task Force for the diagnosis and management of atrial fibrillation of the European Society of Cardiology (ESC) Developed with the special contribution of the European Heart Rhythm Association (EHRA) of the ESC. Eur Heart J.

[29] Romiti GF, Pastori D, Rivera-Caravaca JM (2022). Adherence to the “Atrial Fibrillation Better Care” Pathway in Patients with Atrial Fibrillation: Impact on Clinical Outcomes-A Systematic Review and Meta-Analysis of 285,000 Patients. Thromb Haemost.

[30] Proietti M, Romiti GF, Olshansky B (2018). Improved Outcomes by Integrated Care of Anticoagulated Patients with Atrial Fibrillation Using the Simple ABC (Atrial Fibrillation Better Care) Pathway. Am J Med.

[31] Luo X, Xu W, Ming W-K (2022). Cost-Effectiveness of Mobile Health-Based Integrated Care for Atrial Fibrillation: Model Development and Data Analysis. J Med Internet Res.

[32] Ashenden R, Silagy C, Weller D (1997). A systematic review of the effectiveness of promoting lifestyle change in general practice. Fam Pract.

[33] Joseph-Shehu EM, Ncama BP, Mooi N (2019). The use of information and communication technologies to promote healthy lifestyle behaviour: a systematic scoping review. BMJ Open.

[34] Anand TN, Joseph LM, Geetha AV (2019). Task sharing with non-physician health-care workers for management of blood pressure in low-income and middle-income countries: a systematic review and meta-analysis. Lancet Glob Health.

[35] Ibrahim H, Munkhbayar U, Toivgoo A (2019). Can universal patient-held health booklets promote continuity of care and patient-centred care in low-resource countries? The case of Mongolia. BMJ Qual Saf.

[36] United Nations (2020). World economic situation and prospects.

[37] Nagai M, Abraham S, Okamoto M (2007). Reconstruction of health service systems in the post-conflict Northern Province in Sri Lanka. Health Policy.

[38] Karunathilake IM (2012). Health changes in Sri Lanka: benefits of primary health care and public health. Asia Pac J Public Health.

[39] Moucheraud C, Guo H, Macinko J (2021). Trust In Governments And Health Workers Low Globally, Influencing Attitudes Toward Health Information, Vaccines: Study examines changes in public trust in governments, health workers, and attitudes toward vaccines. Health Aff.

[40] Gilson L (2006). Trust in health care: theoretical perspectives and research needs. J Health Organ Manag.

[41] Organization WH (2020). Pulse survey on continuity of essential health services during the covid-19 pandemic: interim report.

[42] MacVinish S, van Leeuwen C, Hoetjes M (2023). Lessons identified from initiating a thalassaemia programme in a conflict setting: a case study from northeast Syria. Confl Health.

