# Supplementary material for: Inefficiencies identified in healthcare professional-to-patient handover practices for atrial fibrillation: a mixed-methods study in Brazil, China and Sri Lanka
Source: BMJ Glob Health. 2025 Nov 23;10(11):e017517. doi: 10.1136/bmjgh-2024-017517 (PMC12645638; doi:10.1136/bmjgh-2024-017517)
Supplement: online supplemental file 1 [file bmjgh-10-11-s001.docx]

**Supplementary materials:**

Table 1. Quantitative analysis. Between-country analysis on patient and healthcare professional behaviour for effective handover

| Outcome | | Brazil  N=267 | China  N= 298 | Sri Lanka  N=151 | P-value |
| --- | --- | --- | --- | --- | --- |
| Patient behaviour | Did you bring a list of your medicines with you? |  |  |  |  |
|  | Yes | 151 (56.8) | 201 (69.8) | 151 (100) | 0.001^a^ |
|  | No (I forgot it or I had not been given one) | 115 (43.2) | 87 (30.2) | 0 (0) |  |
|  | Excluded (missing or not relevant) | 1 | 10 | 0 |  |
|  | Did you bring your medications with you? |  |  |  |  |
|  | Yes | 46 (17.3) | 168 (58.3) | 25 (16.6) | <0.001 |
|  | No | 220 (82.7) | 120 (41.7) | 126 (83.4) |  |
|  | Excluded (missing or not relevant) | 1 | 10 | 0 |  |
|  | Did you bring any document given to you previously? |  |  |  |  |
|  | Yes | 117 (44.3) | 193 (64.8) | 134 (88.7) | <0.001 |
|  | No (I forgot it or I had not been given one) | 147 (55.7) | 105 (35.2) | 17 (11.3) |  |
|  | Excluded (missing or not relevant) | 3 | 0 | 0 |  |
|  | Do you use your documents at home? |  |  |  |  |
|  | Yes | 256 (96.6) | 272 (94.4) | 111 (73.5) | <0.001 |
|  | No | 9 (3.4) | 16 (5.6) | 40 (26.5) |  |
|  | Excluded (missing or not relevant) | 2 | 10 | 0 |  |
|  | Do you think written documents are important? |  |  |  |  |
|  | Yes | 196 (73.7) | 266 (95.0) | 136 (90.1) | <0.001 |
|  | No/Don’t know | 70 (26.3) | 14 (5.0) | 15 (9.9) |  |
|  | Excluded (missing or not relevant) | 1 | 18 | 0 |  |
| Healthcare professional behaviour | Did the doctor use the document you brought? |  |  |  |  |
|  | Yes | 108 (92.3) | 178 (95.2) | 120 (89.6) | 0.158 |
|  | No/Don’t know | 9 (7.7) | 9 (4.8) | 14 (10.4) |  |
|  | Excluded (missing or not relevant) | 150 | 111 | 17 |  |
|  | Did the doctor write in your documents? |  |  |  |  |
|  | Yes | 57 (48.7) | 172 (95.6) | 70 (52.2) | <0.001 |
|  | No/Don’t know | 60 (51.3) | 8 (4.4) | 64 (47.8) |  |
|  | Excluded (missing or not relevant) | 150 | 118 | 17 |  |
|  | Did the doctor explain how to take care of yourself? |  |  |  |  |
|  | Yes, very well | 187 (73.1) | 210 (79.6) | 108 (71.5) | <0.001 |
|  | Yes, but not very well | 47 (18.4) | 49 (18.6) | 40 (26.5) |  |
|  | No | 22 (8.6) | 5 (1.9) | 3 (2.0) |  |
|  | Excluded (missing or not relevant) | 11 | 34 | 0 |  |

^a^ Sri Lanka was excluded from the analysis.

Table 2. Quantitative analysis. Brazil patient behaviour outcomes; differences among sub-groups of people with AF

| Participant sub-group | Did you bring a list of your medicines with you? | | | Did you bring your medications with you? | | | Did you bring any document given to you previously? | | | Do you use your documents at home? | | | Do you think written documents are important? | | |
| --- | --- | --- | --- | --- | --- | --- | --- | --- | --- | --- | --- | --- | --- | --- | --- |
|  | Yes | No | p-value | Yes | No | p-value | Yes | No | p-value | Yes | No | p-value | Yes | No | p-value |
| Age |  |  |  |  |  |  |  |  |  |  |  |  |  |  |  |
| <70 years | 81 (66.9) | 40 (33.1) | **0.002** | 26 (21.5) | 95 (78.5) | 0.098 | 63 (52.1) | 58 (47.9) | **0.020** | 118 (96.7) | 4 (3.3) | 0.922 | 91 (75.2) | 30 (24.8) | 0.606 |
| 70+ years | 70 (48.3) | 75 (51.7) |  | 20 (13.8) | 125 (86.2) |  | 54 (37.8) | 89 (62.2) |  | 138 (96.5) | 5 (3.5) |  | 105 (72.4) | 40 (27.6) |  |
| Sex |  |  |  |  |  |  |  |  |  |  |  |  |  |  |  |
| Male | 72 (53.3) | 63 (46.7) | 0.251 | 21 (15.6) | 114 (84.4) | 0.447 | 54 (40.3) | 80 (59.7) | 0.182 | 128 (95.5) | 6 (4.5) | 0.326 | 96 (71.1) | 39 (28.9) | 0.333 |
| Female | 79 (60.3) | 52 (39.7) |  | 25 (19.1) | 106 (80.9) |  | 63 (48.5) | 67 (51.5) |  | 128 (97.7) | 3 (2.3) |  | 100 (76.3) | 31 (23.7) |  |
| Education |  |  |  |  |  |  |  |  |  |  |  |  |  |  |  |
| No education | 49 (57.7) | 36 (42.4) | 0.905 | 14 (16.5) | 71 (83.5) | 0.908 | 42 (49.4) | 43 (50.6) | 0.561 | 81 (95.3) | 4 (4.7) | 0.409 | 64 (74.4) | 22 (25.6) | 0.782 |
| Primary | 55 (60.4) | 36 (39.6) |  | 17 (18.7) | 74 (81.3) |  | 41 (45.1) | 50 (55.0) |  | 86 (95.6) | 4 (4.4) |  | 66 (72.5) | 25 (27.5) |  |
| Secondary or higher | 46 (57.5) | 34 (42.5) |  | 15 (18.8) | 65 (81.3) |  | 32 (41.0) | 46 (59.0) |  | 79 (98.8) | 1 (1.2) |  | 61 (77.2) | 18 (22.8) |  |
| Employment |  |  |  |  |  |  |  |  |  |  |  |  |  |  |  |
| Employed | 28 (65.1) | 15 (34.9) | 0.083 | 10 (23.3) | 33 (76.7) | 0.292 | 25 (58.1) | 18 (41.9) | 0.054 | 40 (90.9) | 4 (9.1) | 0.073 | 35 (81.4) | 8 (18.6) | 0.443 |
| Unemployed | 28 (68.3) | 13 (31.7) |  | 9 (22.0) | 32 (78.1) |  | 21 (51.2) | 20 (48.8) |  | 39 (97.5) | 1 (2.5) |  | 29 (70.7) | 12 (29.3) |  |
| Retired | 95 (52.2) | 87 (47.8) |  | 27 (14.8) | 155 (85.2) |  | 71 (39.4) | 109 (60.6) |  | 177 (97.8) | 4 (2.2) |  | 132 (72.5) | 50 (27.5) |  |
| Marital status |  |  |  |  |  |  |  |  |  |  |  |  |  |  |  |
| Single | 60 (59.4) | 41 (40.6) | 0.586 | 13 (12.9) | 88 (87.1) | 0.104 | 40 (39.6) | 61 (60.4) | 0.198 | 97 (96.0) | 4 (4.0) | 0.327 | 76 (75.3) | 25 (24.7) | 0.765 |
| Married | 89 (56.0) | 70 (44.0) |  | 33 (20.8) | 126 (79.3) |  | 75 (47.8) | 82 (52.2) |  | 153 (96.8) | 5 (3.2) |  | 117 (73.6) | 42 (26.4) |  |
| AF duration |  |  |  |  |  |  |  |  |  |  |  |  |  |  |  |
| <5 years | 59 (65.6) | 31 (34.4) | 0.323 | 14 (15.6) | 76 (84.4) | 0.523 | 45 (50.6) | 44 (49.4) | 0.785 | 88 (97.8) | 2 (2.2) | 0.739 | 70 (78.7) | 19 (21.3) | 0.768 |
| 5-10 years | 38 (55.9) | 30 (44.1) |  | 12 (17.7) | 56 (82.4) |  | 32 (46.4) | 37 (53.6) |  | 66 (95.7) | 3 (4.3) |  | 51 (73.9) | 18 (26.1) |  |
| >10 years | 45 (55.6) | 36 (44.4) |  | 18 (22.2) | 63 (77.8) |  | 36 (45.6) | 43 (54.4) |  | 77 (96.3) | 3 (3.7) |  | 61 (75.3) | 20 (24.7) |  |
| OAC use |  |  |  |  |  |  |  |  |  |  |  |  |  |  |  |
| Warfarin | 127 (55.7) | 101 (44.3) | 0.529 | 37 (16.2) | 191 (83.8) | 0.159 | 99 (44.0) | 126 (56.0) | 0.642 | 219 (96.5) | 8 (3.5) | 0.718 | 172 (75.4) | 56 (24.6) | 0.260 |
| NOACs | 9 (56.3) | 7 (43.8) |  | 2 (12.5) | 14 (87.5) |  | 6 (37.5) | 10 (62.5) |  | 16 (100) | 0 (0) |  | 10 (66.7) | 5 (33.3) |  |
| No OACs | 15 (68.2) | 7 (31.8) |  | 7 (31.8) | 15 (68.2) |  | 12 (52.3) | 11 (47.8) |  | 21 (95.5) | 1 (4.5) |  | 14 (60.9) | 9 (39.1) |  |

Table 3. Quantitative analysis. Brazil healthcare professional behaviour outcomes; differences among sub-groups of people with AF

| Participant sub-group | Did the doctor use the document you brought? | | | Did the doctor write in your documents? | | | Did the doctor explain how to take care of yourself? | | | |
| --- | --- | --- | --- | --- | --- | --- | --- | --- | --- | --- |
|  | Yes | No/don’t know | p-value | Yes | No/don’t know | p-value | Yes, very well | Yes, not very well | No | p-value |
| Age |  |  |  |  |  |  |  |  |  |  |
| <70 years | 61 (96.8) | 2 (3.2) | **0.048** | 28 (44.4) | 35 (55.6) | 0.318 | 91 (77.8) | 17 (14.5) | 9 (7.7) | 0.275 |
| 70+ years | 47 (87.0) | 7 (13.0) |  | 29 (53.7) | 25 (46.3) |  | 96 (69.1) | 30 (21.6) | 13 (9.4) |  |
| Sex |  |  |  |  |  |  |  |  |  |  |
| Male | 50 (92.6) | 4 (7.4) | 0.915 | 28 (51.9) | 26 (48.2) | 0.530 | 96 (73.9) | 22 (16.9) | 12 (9.2) | 0.801 |
| Female | 58 (92.1) | 5 (7.9) |  | 29 (46.0) | 34 (54.0) |  | 91 (72.2) | 25 (19.8) | 10 (7.9) |  |
| Education |  |  |  |  |  |  |  |  |  |  |
| No education | 40 (95.2) | 36 (42.4) | 0.733 | 20 (47.6) | 22 (52.4) | 0.986 | 61 (73.5) | 14 (16.9) | 8 (9.6) | 0.264 |
| Primary | 37 (90.2) | 36 (39.6) |  | 20 (48.8) | 21 (51.2) |  | 57 (66.3) | 19 (22.1) | 10 (11.6) |  |
| Secondary or higher | 29 (90.6) | 34 (42.5) |  | 15 (46.9) | 17 (53.1) |  | 62 (80.5) | 12 (15.6) | 3 (3.9) |  |
| Employment |  |  |  |  |  |  |  |  |  |  |
| Employed | 23 (92.0) | 2 (8.0) | 0.854 | 12 (48.0) | 13 (52.0) | 0.527 | 34 (77.3) | 7 (15.9) | 3 (6.8) | 0.697 |
| Unemployed | 20 (95.2) | 1 (4.8) |  | 8 (38.1) | 13 (61.9) |  | 25 (64.1) | 9 (23.1) | 5 (12.8) |  |
| Retired | 65 (91.6) | 6 (8.5) |  | 37 (52.1) | 34 (47.9) |  | 128 (74.0) | 31 (17.9) | 14 (8.1) |  |
| Marital status |  |  |  |  |  |  |  |  |  |  |
| Single | 38 (95.0) | 2 (5.0) | 0.410 | 22 (55.0) | 18 (56.0) | 0.261 | 73 (74.5) | 18 (18.4) | 7 (7.1) | 0.853 |
| Married | 68 (90.7) | 7 (9.3) |  | 33 (44.0) | 42 (56.0) |  | 111 (72.6) | 28 (18.3) | 14 (9.2) |  |
| AF duration |  |  |  |  |  |  |  |  |  |  |
| <5 years | 43 (95.6) | 2 (4.4) | 0.513 | 23 (51.1) | 22 (48.9) | 0.933 | 58 (67.4) | 19 (22.1) | 9 (10.5) | 0.180 |
| 5-10 years | 29 (90.6) | 3 (9.4) |  | 15 (46.9) | 17 (53.1) |  | 48 (71.6) | 16 (23.9) | 3 (4.5) |  |
| >10 years | 32 (88.9) | 4 (11.1) |  | 18 (50.0) | 18 (50.0) |  | 63 (79.8) | 9 (11.4) | 7 (8.9) |  |
| OAC use |  |  |  |  |  |  |  |  |  |  |
| Warfarin | 91 (91.9) | 8 (8.1) | 0.427 | 50 (50.5) | 49 (49.5) | 0.531 | 168 (74.3) | 38 (16.8) | 20 (8.9) | 0.506 |
| NOACs | 5 (83.3) | 1 (16.7) |  | 3 (50.0) | 3 (50.0) |  | 10 (66.7) | 4 (26.7) | 1 (6.7) |  |
| No OACs | 12 (100) | 0 (0) |  | 4 (33.3) | 8 (66.7) |  | 9 (60.0) | 5 (33.3) | 1 (6.7) |  |

Table 4. Quantitative analysis. China patient behaviour outcomes; differences among sub-groups of people with AF

| Participant sub-group | Did you bring a list of your medicines with you? | | | Did you bring your medications with you? | | | Did you bring any document given to you previously? | | | Do you use your documents at home? | | | Do you think written documents are important? | | |
| --- | --- | --- | --- | --- | --- | --- | --- | --- | --- | --- | --- | --- | --- | --- | --- |
|  | Yes | No | p-value | Yes | No | p-value | Yes | No | p-value | Yes | No | p-value | Yes | No | p-value |
| Age |  |  |  |  |  |  |  |  |  |  |  |  |  |  |  |
| <70 years | 129 (70.1) | 55 (29.9) | 0.876 | 111 (60.3) | 73 (39.7) | 0.362 | 125 (64.8) | 68 (35.2) | 0.999 | 176 (93.6) | 12 (6.4) | 0.401 | 174 (96.1) | 7 (3.9) | 0.240 |
| 70+ years | 72 (69.2) | 32 (30.8) |  | 57 (54.8) | 47 (45.2) |  | 68 (64.8) | 37 (35.2) |  | 96 (96.0) | 4 (4.0) |  | 92 (92.9) | 7 (3.9) |  |
| Sex |  |  |  |  |  |  |  |  |  |  |  |  |  |  |  |
| Male | 123 (69.1) | 63 (46.7) | 0.265 | 101 (56.7) | 77 (43.3) | 0.486 | 120 (64.9) | 65 (35.1) | 0.963 | 164 (92.1) | 14 (7.9) | **0.030** | 168 (97.7) | 4 (2.3) | **0.010** |
| Female | 78 (70.9) | 52 (39.7) |  | 67 (60.9) | 43 (39.1) |  | 73 (64.6) | 40 (35.4) |  | 108 (98.2) | 2 (1.8) |  | 98 (90.7) | 10 (9.3) |  |
| Education |  |  |  |  |  |  |  |  |  |  |  |  |  |  |  |
| No education | 11 (73.3) | 4 (26.7) | 0.235 | 12 (80.0) | 3 (20.0) | 0.080 | 10 (55.6) | 8 (44.4) | 0.101 | 17 (100) | 0 (0) | 0.507 | 17 (94.4) | 1 (5.6) | 0.984 |
| Primary | 15 (55.6) | 12 (44.4) |  | 12 (44.4) | 15 (55.6) |  | 13 (48.2) | 14 (51.9) |  | 22 (91.7) | 2 (8.3) |  | 22 (95.7) | 1 (4.3) |  |
| Secondary or higher | 175 (71.1) | 71 (28.9) |  | 144 (58.5) | 102 (41.5) |  | 170 (67.2) | 83 (32.8) |  | 233 (94.3) | 14 (5.7) |  | 227 (95.0) | 12 (5.0) |  |
| Employment |  |  |  |  |  |  |  |  |  |  |  |  |  |  |  |
| Employed | 45 (75.0) | 15 (25.0) | 0.295 | 38 (63.3) | 22 (36.7) | 0.636 | 41 (65.1) | 22 (34.9) | 0.670 | 55 (88.7) | 7 (11.3) | 0.069 | 59 (100) | 0 (0) | **0.016** |
| Unemployed | 13 (81.3) | 3 (18.8) |  | 9 (56.3) | 7 (43.8) |  | 14 (73.7) | 5 (26.3) |  | 18 (100) | 0 (0) |  | 15 (83.3) | 3 (16.7) |  |
| Retired | 139 (67.2) | 68 (32.9) |  | 117 (56.5) | 90 (43.5) |  | 134 (63.5) | 77 (36.5) |  | 194 (95.6) | 9 (4.4) |  | 187 (94.4) | 11 (5.6) |  |
| Marital status |  |  |  |  |  |  |  |  |  |  |  |  |  |  |  |
| Single | 6 (75.0) | 2 (25.0) | 0.781 | 3 (37.5) | 5 (62.5) | 0.195 | 6 (60.0) | 4 (40.0) | 0.691 | 9 (90.0) | 1 (10.0) | 0.507 | 9 (100) | 0 (0) | 0.477 |
| Married | 193 (71.0) | 79 (29.0) |  | 164 (60.3) | 108 (39.7) |  | 185 (66.1) | 95 (33.9) |  | 256 (94.8) | 14 (5.2) |  | 249 (94.7) | 14 (5.3) |  |
| AF duration |  |  |  |  |  |  |  |  |  |  |  |  |  |  |  |
| <5 years | 141 (72.3) | 54 (27.7) | 0.613 | 116 (59.5) | 79 (40.5) | 0.624 | 133 (65.5) | 70 (34.5) | 0.419 | 188 (95.0) | 10 (5.0) | 0.348 | 187 (95.9) | 8 (4.1) | 0.473 |
| 5-10 years | 25 (69.4) | 11 (30.6) |  | 23 (63.9) | 13 (36.1) |  | 26 (70.3) | 11 (29.7) |  | 37 (100) | 0 (0) |  | 35 (100) | 0 (0) |  |
| >10 years | 27 (79.4) | 7 (20.6) |  | 23 (67.7) | 11 (32.4) |  | 26 (76.5) | 8 (23.5) |  | 30 (93.8) | 2 (6.2) |  | 29 (96.7) | 1 (3.3) |  |
| OAC use |  |  |  |  |  |  |  |  |  |  |  |  |  |  |  |
| Warfarin | 47 (78.3) | 13 (21.7) | **0.042** | 47 (78.3) | 13 (21.7) | **0.002** | 46 (75.4) | 15 (24.6) | 0.100 | 57 (96.6) | 2 (3.4) | 0.285 | 55 (94.8) | 3 (5.2) | 0.957 |
| NOACs | 86 (62.8) | 51 (37.2) |  | 75 (54.7) | 62 (45.3) |  | 83 (59.7) | 56 (40.3) |  | 130 (95.6) | 6 (4.4) |  | 125 (94.7) | 7 (5.3) |  |
| No OACs | 68 (74.7) | 23 (25.3) |  | 46 (50.6) | 45 (49.5) |  | 64 (65.3) | 34 (34.7) |  | 85 (91.4) | 8 (8.6) |  | 86 (95.6) | 4 (4.4) |  |

Table 5. Quantitative analysis. China healthcare professional behaviour outcomes; differences among sub-groups of people with AF

| Participant sub-group | Did the doctor use the document you brought? | | | Did the doctor write in your documents? | | | Did the doctor explain how to take care of yourself? | | | |
| --- | --- | --- | --- | --- | --- | --- | --- | --- | --- | --- |
|  | Yes | No/don’t know | p-value | Yes | No/don’t know | p-value | Yes, very well | Yes, not very well | No | p-value |
| Age |  |  |  |  |  |  |  |  |  |  |
| <70 years | 115 (95.0) | 6 (5.0) | 0.900 | 113 (94.2) | 7 (5.8) | 0.201 | 137 (78.7) | 32 (18.4) | 5 (2.9) | 0.993 |
| 70+ years | 63 (95.5) | 3 (4.6) |  | 59 (98.3) | 1 (1.7) |  | 73 (81.1) | 17 (18.9) | 0 (0) |  |
| Sex |  |  |  |  |  |  |  |  |  |  |
| Male | 107 (93.0) | 8 (7.0) | 0.083 | 107 (94.7) | 6 (5.3) | 0.464 | 126 (77.8) | 32 (19.8) | 4 (2.5) | 0.493 |
| Female | 71 (98.6) | 1 (1.14) |  | 65 (97.0) | 2 (3.0) |  | 84 (82.3) | 17 (16.7) | 1 (1.0) |  |
| Education |  |  |  |  |  |  |  |  |  |  |
| No education | 9 (90.0) | 1 (10.0) | 0.629 | 9 (90.0) | 1 (10.0) | 0.526 | 14 (87.5) | 2 (12.5) | 0 (0) | 0.727 |
| Primary | 12 (92.3) | 1 (7.7) |  | 12 (100) | 0 (0) |  | 17 (77.3) | 5 (22.7) | 0 (0) |  |
| Secondary or higher | 157 (95.7) | 7 (4.3) |  | 151 (95.6) | 7 (4.4) |  | 179 (79.2) | 42 (18.6) | 5 (2.2) |  |
| Employment |  |  |  |  |  |  |  |  |  |  |
| Employed | 40 (100) | 0 (0) | 0.263 | 39 (100) | 0 (0) | 0.181 | 46 (83.6) | 6 (10.9) | 3 (5.5) | 0.235 |
| Unemployed | 13 (92.9) | 1 (7.1) |  | 12 (100) | 0 (0) |  | 13 (86.7) | 2 (13.3) | 0 (0) |  |
| Retired | 121 (93.8) | 8 (6.2) |  | 117 (93.6) | 8 (6.4) |  | 147 (77.8) | 40 (21.2) | 2 (1.1) |  |
| Marital status |  |  |  |  |  |  |  |  |  |  |
| Single | 5 (100) | 0 (0) | 0.609 | 5 (100) | 0 (0) | 0.624 | 6 (75.0) | 2 (25.0) | 0 (0) | 0.611 |
| Married | 172 (95.0) | 9 (5.0) |  | 166 (95.4) | 8 (4.6) |  | 201 (80.4) | 44 (17.6) | 5 (2.0) |  |
| AF duration |  |  |  |  |  |  |  |  |  |  |
| <5 years | 124 (96.1) | 5 (3.9) | 0.604 | 122 (95.3) | 6 (4.7) | 0.427 | 150 (82.4) | 27 (14.8) | 5 (2.8) | 0.536 |
| 5-10 years | 24 (96.0) | 1 (4.0) |  | 24 (92.3) | 2 (7.7) |  | 27 (77.1) | 8 (22.9) | 0 (0) |  |
| >10 years | 25 (100) | 0 (0) |  | 23 (100) | 0 (0) |  | 27 (84.4) | 5 (15.6) | 0 (0) |  |
| OAC use |  |  |  |  |  |  |  |  |  |  |
| Warfarin | 42 (95.5) | 2 (4.6) | 0.319 | 44 (97.8) | 1 (2.2) | 0.492 | 52 (94.6) | 3 (5.4) | 0 (0) | **0.002** |
| NOACs | 78 (97.5) | 2 (2.5) |  | 72 (93.5) | 5 (6.5) |  | 92 (71.9) | 34 (26.6) | 2 (1.6) |  |
| No OACs | 58 (92.1) | 5 (7.9) |  | 56 (96.6) | 2 (3.4) |  | 66 (81.5) | 12 (14.8) | 3 (3.7) |  |

Table 6. Quantitative analysis. Sri Lanka patient behaviour outcomes; differences among sub-groups of people with AF

| Participant sub-group | Did you bring your medications with you? | | | Did you bring any document given to you previously? | | | Do you use your documents at home? | | | Do you think written documents are important? | | |
| --- | --- | --- | --- | --- | --- | --- | --- | --- | --- | --- | --- | --- |
|  | Yes | No | p-value | Yes | No | p-value | Yes | No | p-value | Yes | No | p-value |
| Age |  |  |  |  |  |  |  |  |  |  |  |  |
| <70 years | 21 (16.5) | 106 (83.5) | 0.987 | 111 (87.4) | 16 (12.6) | 0.231 | 95 (74.8) | 32 (25.2) | 0.407 | 116 (91.3) | 11 (8.7) | 0.229 |
| 70+ years | 4 (16.7) | 20 (83.3) |  | 23 (95.8) | 1 (4.2) |  | 16 (66.7) | 8 (33.3) |  | 20 (83.3) | 4 (16.7) |  |
| Sex |  |  |  |  |  |  |  |  |  |  |  |  |
| Male | 9 (20.0) | 36 (80.0) | 0.458 | 42 (93.3) | 3 (6.7) | 0.245 | 34 (75.6) | 11 (24.4) | 0.711 | 40 (88.9) | 5 (11.1) | 0.753 |
| Female | 16 (15.1) | 90 (84.9) |  | 92 (86.8) | 14 (13.2) |  | 77 (72.6) | 29 (27.4) |  | 96 (90.6) | 10 (9.4) |  |
| Education |  |  |  |  |  |  |  |  |  |  |  |  |
| No education | 3 (13.6) | 19 (86.4) | 0.136 | 19 (86.4) | 3 (13.6) | 0.859 | 16 (72.7) | 6 (27.3) | 0.969 | 20 (90.9) | 2 (9.1) | 0.429 |
| Primary | 3 (7.5) | 37 (92.5) |  | 35 (87.5) | 5 (12.5) |  | 30 (75.0) | 10 (25.0) |  | 38 (95.0) | 2 (5.0) |  |
| Secondary or higher | 19 (21.4) | 70 (78.7) |  | 80 (89.9) | 9 (10.1) |  | 65 (73.0) | 24 (27.0) |  | 78 (87.6) | 11 (12.4) |  |
| Employment |  |  |  |  |  |  |  |  |  |  |  |  |
| Employed | 7 (25.9) | 20 (74.1) | 0.271 | 24 (88.9) | 3 (11.1) | 0.450 | 23 (85.2) | 4 (14.8) | 0.192 | 25 (92.6) | 2 (7.4) | 0.837 |
| Unemployed | 16 (14.7) | 93 (85.3) |  | 96 (88.1) | 13 (11.9) |  | 76 (69.7) | 33 (30.3) |  | 97 (89.0) | 12 (11.0) |  |
| Retired | 1 (8.3) | 11 (91.7) |  | 12 (100) | 0 (0) |  | 10 (83.3) | 2 (16.7) |  | 11 (91.7) | 1 (8.3) |  |
| Marital status |  |  |  |  |  |  |  |  |  |  |  |  |
| Single | 9 (15.8) | 48 (84.2) | 0.844 | 50 (87.7) | 7 (12.3) | 0.757 | 37 (64.9) | 20 (35.1) | 0.062 | 49 (86.0) | 8 (14.0) | 0.190 |
| Married | 16 (17.0) | 78 (83.0) |  | 84 (89.4) | 10 (10.6) |  | 74 (78.7) | 20 (21.3) |  | 87 (92.6) | 7 (7.4) |  |
| AF duration |  |  |  |  |  |  |  |  |  |  |  |  |
| <5 years | 17 (16.2) | 88 (83.8) | 0.856 | 94 (89.5) | 11 (10.5) | 0.816 | 74 (70.5) | 31 (29.5) | 0.329 | 95 (90.5) | 10 (9.5) | 0.857 |
| 5-10 years | 6 (20.0) | 24 (80.0) |  | 26 (86.7) | 4 (13.3) |  | 24 (80.0) | 6 (20.0) |  | 27 (90.0) | 3 (10.0) |  |
| >10 years | 2 (14.3) | 12 (85.7) |  | 13 (92.9) | 1 (7.1) |  | 12 (85.7) | 2 (14.3) |  | 12 (85.7) | 2 (14.3) |  |

Question ‘Did you bring a list of your medicines with you?’ was excluded due to 100% saying yes to this question.

Sub-group ‘OAC use’ was excluded due to all but one participant being on warfarin.

Table 7. Quantitative analysis. Sri Lanka healthcare professional behaviour outcomes; differences among sub-groups of people with AF

| Participant sub-group | Did the doctor use the document you brought? | | | Did the doctor write in your documents? | | | Did the doctor explain how to take care of yourself? | | | |
| --- | --- | --- | --- | --- | --- | --- | --- | --- | --- | --- |
|  | Yes | No/don’t know | p-value | Yes | No/don’t know | p-value | Yes, very well | Yes, not very well | No | p-value |
| Age |  |  |  |  |  |  |  |  |  |  |
| <70 years | 99 (89.2) | 12 (10.8) | 0.763 | 58 (52.3) | 53 (47.8) | 0.995 | 94 (74.0) | 31 (24.4) | 2 (1.6) | 0.262 |
| 70+ years | 21 (91.3) | 2 (8.7) |  | 12 (52.2) | 11 (47.8) |  | 14 (58.3) | 9 (37.5) | 1 (4.2) |  |
| Sex |  |  |  |  |  |  |  |  |  |  |
| Male | 36 (85.7) | 6 (14.3) | 0.326 | 21 (50.0) | 21 (50.0) | 0.726 | 36 (80.0) | 8 (17.8) | 1 (2.2) | 0.287 |
| Female | 84 (91.3) | 8 (8.7) |  | 49 (53.3) | 43 (46.7) |  | 72 (67.9) | 32 (30.2) | 2 (1.9) |  |
| Education |  |  |  |  |  |  |  |  |  |  |
| No education | 17 (89.5) | 2 (10.5) | 0.549 | 11 (57.9) | 8 (42.1) | 0.793 | 16 (72.7) | 6 (27.3) | 0 (0) | 0.599 |
| Primary | 33 (94.3) | 2 (5.7) |  | 19 (54.3) | 16 (45.7) |  | 27 (67.5) | 11 (27.5) | 2 (5.0) |  |
| Secondary or higher | 70 (87.5) | 10 (12.5) |  | 40 (50.0) | 40 (50.0) |  | 65 (73.0) | 23 (25.8) | 1 (1.1) |  |
| Employment |  |  |  |  |  |  |  |  |  |  |
| Employed | 21 (87.5) | 3 (12.5) | 0.885 | 11 (45.8) | 13 (54.2) | 0.740 | 23 (85.2) | 4 (14.8) | 0 (0) | 0.214 |
| Unemployed | 87 (90.6) | 9 (9.4) |  | 51 (53.1) | 45 (46.9) |  | 74 (67.9) | 33 (30.3) | 2 (1.8) |  |
| Retired | 11 (91.7) | 1 (8.3) |  | 7 (58.3) | 5 (41.7) |  | 8 (66.7) | 3 (25.0) | 1 (8.3) |  |
| Marital status |  |  |  |  |  |  |  |  |  |  |
| Single | 43 (86.0) | 7 (14.0) | 0.300 | 23 (46.0) | 27 (54.0) | 0.265 | 39 (68.4) | 16 (28.1) | 2 (3.5) | 0.528 |
| Married | 77 (91.7) | 7 (8.3) |  | 47 (56.0) | 37 (44.0) |  | 69 (73.4) | 24 (25.5) | 1 (1.1) |  |
| AF duration |  |  |  |  |  |  |  |  |  |  |
| <5 years | 82 (87.2) | 12 (12.8) | 0.324 | 53 (56.4) | 41 (43.6) | 0.213 | 78 (74.3) | 26 (24.8) | 1 (1.0) | 0.266 |
| 5-10 years | 24 (92.3) | 2 (7.7) |  | 13 (50.0) | 13 (50.0) |  | 21 (70.0) | 8 (26.7) | 1 (33.3) |  |
| >10 years | 13 (100) | 0 (0) |  | 4 (30.8) | 9 (69.2) |  | 7 (50.0) | 6 (42.9) | 1 (7.1) |  |

Sub-group ‘OAC use’ was excluded due to all but one participant being on warfarin.


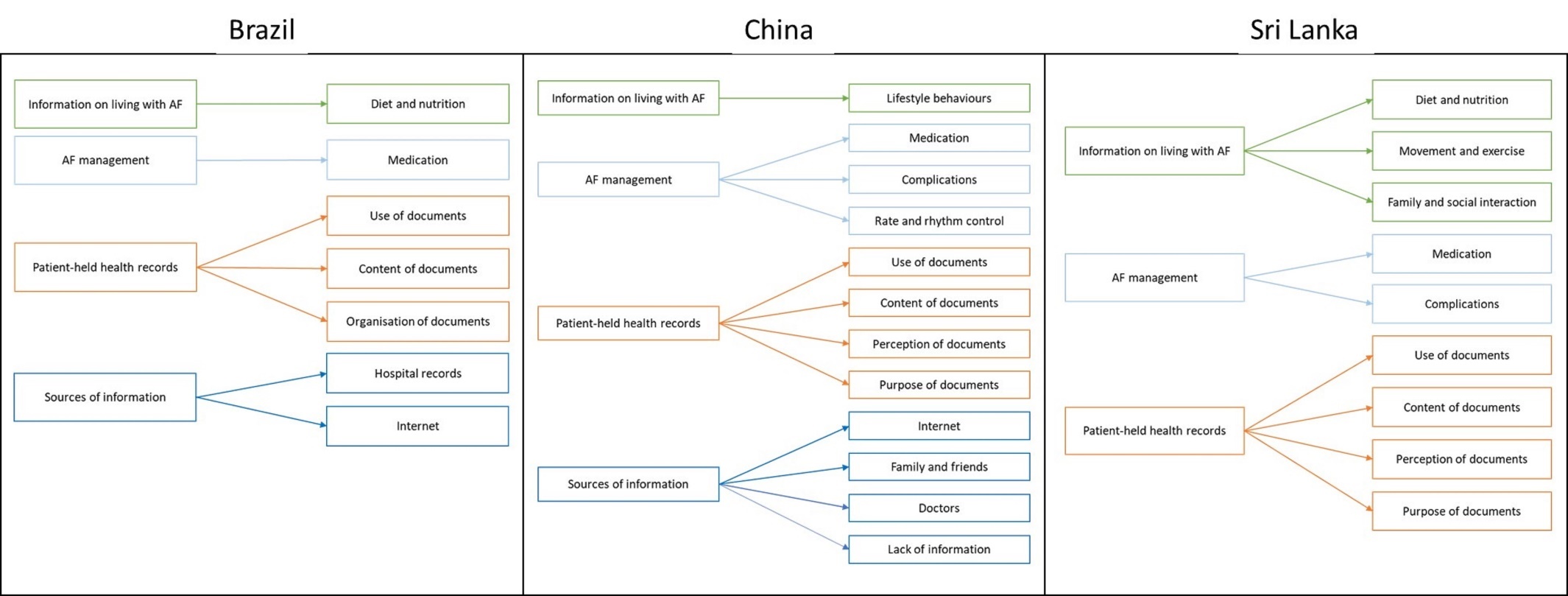


Figure 1. Qualitative analysis. Coding trees of themes and sub-themes for each country.

Themes identified from one country were not automatically applied to the other countries; however, many of the same themes were discovered from each country. Four main themes related to handover were identified from the Brazilian and Chinese FGDs: 1) *information on living with AF,* 2) *AF management,* 3) *patient-held health records* and 4) *sources of information* (Figure 1). The former three themes were also identified as main themes from the Sri Lankan FGDs and were the only themes highlighted.

Table 8. Qualitative analysis. Supporting quotes for each theme and sub-theme.

| Theme | Sub-theme | *Supporting quote* (Country, FGD number, Participant (P) number) |
| --- | --- | --- |
| Information on living with AF | Diet and nutrition | “*I avoid the green leaf more, but I like it, but I avoid it, but I know what is needed, the doctor guided me”* (Brazil, FGD 2, P4)  *“I don't eat anything green because of Warfarin ... not like kale, stuff like that. They say it changes, right? Mine changes if I eat.”* (Brazil, FGD 1, P5)  *“have to be control in food, have to control in taking vegetables as using warfarin tablet, can’t eat unwanted vegetables, even though be in control blood is clotting”* (Sri Lanka, FGD 2, P3)  *“They told not to eat spinach and soya.”* (Sri Lanka, FGD 3, P1) |
|  | Movement and exercise | *“they asked me to walk, I used to walk for two years following the operation.”* (Sri Lanka, FGD 1, P7)  *“I should not lift weights, they told this when using warfarin after the operation.”* (Sri Lanka, FGD 3, P7) |
|  | Family and social interaction | *“Loud vibration is also not good, I ask my family if a funeral happened in my family let me take somewhere to avoid the loud sound and vibration.”* (Sri Lanka, FGD 1, P4)  “Palpitation will increase if hear loud sounds.” (Sri Lanka, FGD 3, P3) |
|  | Lifestyle behaviours | *“Medicine and lifestyle adjustment, drinking water, eating fruit and vegetables, as well as reasonable exercise [can prevent other conditions].”* (China, FGD 7, P1)  *“All I know is that I should take the medicine as prescribed by my doctor. Also I should pay attention to lifestyle.”* (China, FGD 4, P1)  *“Doctors have told me a lot about the disease of atrial fibrillation, including the symptoms of atrial fibrillation, how to reduce the incidence of atrial fibrillation, such as no smoking, no drinking, paying attention to lifestyle, avoiding fatigue, using anticoagulants for treatment and so on.”* (China, FGD 1, P2) |
| AF management | Medication | *“I don't remember [how long I have to take warfarin], I don't think they informed me, I think it's forever.”* (Brazil, FGD 3, P4)  *“They wrote to [consultant cardiologist] again he told to stop warfarin to extract the tooth.”* (Sri Lanka, FGD 2, P1)  *“Doctors have told me about the treatment of AF. [The doctor] told me that atrial fibrillation should be treated with anticoagulation to prevent thrombosis. Oral warfarin is prone to bleeding. Now I am recommended to take Pradaxa as anticoagulant.”* (China, FGD 1, P8)  *“The doctors haven’t explained it to me because the doctor was too busy. But the doctor will prescribe the medicine for me. I read the instructions by myself after I got the medicine.”* (China, FGD 2, P3)  *“I just want to be clear about the question of medication, whether to take it for life long and whether my current medications need adjustments.”* (China, FGD 7, P2) |
|  | Complications | *“We were informed that it will be difficult if get any wound, bleeding won’t stop, in order to control bleeding, seek hospital immediately, need to put ice.”* (Sri Lanka, FGD 1, P4)  *“The doctor told me that I had a serious disease that could form a blood clot and cause a blockage, such as a stroke.”* (China, FGD 5, P1) |
|  | Rate and rhythm control | *“I know that AF can lead to heart failure and can be treated with ablation. I don't know the rest.”* (China, FGD 2, P4)  *“Doctors have introduced to me what is atrial fibrillation, the incidence of atrial fibrillation, harm, and related treatment. Treatment includes heart rate control, anticoagulation, thrombosis prevention and radiofrequency ablation.”* (China, FGD 1, P10) |
| Patient-held health records | Use of documents | *“[The doctor] doesn't give me a report, but he gives me a request that says ‘Take [the medication] so you don't forget’.”* (Brazil, FGD 2, P1)  *“But my God they want to know everything right people? How was the story and you have to have the documents to prove it.”* (Brazil, FGD 2, P5)  *“Yes [the document given to us with INR results is important], because usually the doctor asks and we never know what it is.”* (Brazil, FGD 1, P6)  *“Wherever we go have to carry this clinic book.”* (Sri Lanka, FGD 1, P10)  *“We receive medication by showing this clinic book and informing that we are having this disease.”* (Sri Lanka, FGD 3, P8)  *“they have given [documents] to me. But I lost them long ago.”* (China, FGD 4, P2)  *“I have hard copies of medical records, and I keep them well. [The doctor] will compare the results of the previous examination with the present one.”* (China, FGD 7, P1)  *“Doctors sometimes give me paper medical records, sometimes they don't. I want a paper medical record. It's useful. Paper cases can help me to observe the changes of my condition.”* (China, FGD 1, P4)  *“I have these medical records. The doctor told me to do routine check-ups every three months. No doctors told me to bring my last medical record. I keep the results of every examination, but I don’t bring them with me every time I see a doctor.”* (China, FGD 7, P2)  *“Even if I take [documents to my next appointment], the doctor will not look it patiently. There are so many people waiting to see a doctor. The doctor ended up talking with a few words in several minutes.”* (China, FGD 3, P1) |
|  | Content of documents | *“There are papers they give, but the exam stays there with them. What they give us is the [INR results], we do it and take the document.”* (Brazil, FGD 3, P5)  *“All the test and treatment which were taken are mentioned in this [clinic] book and our phone numbers also present here.”* (Sri Lanka, FGD 1, P8)  *“The doctor gave me a paper medical record, examination results and prescription.”* (China, FGD 1, P8)  *“I have medical records. There is no special content written in the medical record. At most, there are prescriptions. There are also some test sheets, including some indicators like blood lipids. But very few are written by hand with few words.”* (China, FGD 3, P1) |
|  | Organisation of documents | *“I am the king of the archive, I have a box at home that is already huge because I keep everything there. These days I was thinking about taking things 2 years back and throwing them all away, there's no reason for me to keep that, I'm thinking about scanning and putting it in the computer.”* (Brazil, FGD 3, P2)  *“I have a separate folder [for my documents] for the cardiologist, pulmonologist and endocrinologist. it's a way when you need it it´s there. Everything separate. Even by exam dates.”* (Brazil, FGD 1, P5) |
|  | Perception of documents | *“I do think it's important to have because every place we go they ask us to tell our story, but it's no use just talking, they must see everything that happened to us.”* (Brazil, FGD 2, P5)  *“While we were displaced due to war we carried this copy carefully as same as money and jewels.”* (Sri Lanka, FGD 1, P4)  *“[Showing their clinic book] This is the life of all us.”* (Sri Lanka, FGD 2, P2)  *“I don't have any paper cases, but I have a test list and a prescription for drugs. I don't think it's necessary for doctors to give paper cases because computers keep records.”* (China, FGD 1, P7)  *“I don't have any paper cases, but there are prescriptions and laboratory tests. Doctors sometimes give me paper medical records, sometimes they don't. I want a paper medical record. It's useful. Paper cases can help me to observe the changes of my condition.”* (China, FGD 1, P4)  *“It is necessary for a doctor to give a paper medical record. There are too many patients, it is difficult for the doctors to remember everyone's condition.”* (China, FGD 1, P2) |
|  | Purpose of documents | *“Others don’t know our story, by showing her copy…in this book I had all my things from the [consultant cardiologist].”* (Sri Lanka, FGD 2, P3)  *“they gave me like this [laminated piece of paper], they asked to hang it, I always had this in my purse. Because some thing happened in the road others can see this immediately.”* (Sri Lanka, FGD 1, P4)  *“I kept these materials for my own preservation, so that the next doctor can understand my condition. I want to get better treatment with the help of these documents.”* (China, FGD 2, P1) |
| Sources of information | Hospital records | *“If I want the medical report from InCor, I just go there and ask.”* (Brazil, FGD 3, P5)  *“I know that through the computer I can see all the [hospital] results. I access it through the internet and through the Hospital das Clínicas, I access it and get it there, endoscopy, blood test, I get everything."* (Brazil, FGD 2, P4) |
|  | Internet | *“I searched Google [for more information]. Doctor Google. I put how it happened, what it is, what provokes it, what causes it.”* (Brazil, FGD 1, P1)  *“I look for clarification on Google.” (Brazil, FGD 3, P3)*  *“I looked up the information related to medicine on my mobile phone, I think I should be cautious, maybe Traditional Chinese medicine will be better.”* (China, FGD 6, P3)  *“I have searched information on the Internet by myself using the Baidu website. I learned that atrial fibrillation can cause blood clots.”* (China, FGD 2, P1) |
|  | Family and friends | *“I also communicated with my fellow patients. I have five or six colleagues with atrial fibrillation in my company, and they are all taking warfarin, they are old, and they have not had surgery.”* (China, FGD 7, P4)  *“I didn't know there was a [risk of] blood clot. My son once told me that AF can cause blood clots.”* (China, FGD 3, P2) |
|  | Doctors | *“I will ask a doctor for help. I won’t search the information online when I have a problem or a change of my condition. I don't think the information online is accurate. Atrial fibrillation is not as simple as other disease like headaches and colds. For example, the left atrial appendage, such a small and useless place, cannot be explained online.”* (China, FGD 4, P2)  *“After I was discharged from the railway hospital, I did not know what atrial fibrillation was. I asked a doctor in Beijing.”* (China, FGD 6, P4) |
|  | Lack of information | *“Due to my low educational background, I can't use the Internet or books to look up relevant information.” (China, FGD 5, P1)*  *“Because I have to take care of my wife every day, I don't have the time and energy to find information about atrial fibrillation by myself.” (China, FGD 5, P2)*  *“I want to communicate with people who is knowledgeable about AF but I don't have such a good friend.” (China, FGD 2, P3)* |
